# Pylorus-spanning diffuse gastric lipomatosis as an uncommon cause of intermittent gastric outlet obstruction: a case report

**DOI:** 10.1097/RC9.0000000000000278

**Published:** 2026-02-12

**Authors:** Kyeong Woon Choi

**Affiliations:** Department of Surgery, Ilsan Paik Hospital, Inje University College of Medicine, Goyang, Republic of Korea

**Keywords:** distal gastrectomy, gastric lipoma, gastric lipomatosis, gastric outlet obstruction, laparoscopy, pyloric channel

## Abstract

**Introduction and importance:**

Gastric lipomas are uncommon; diffuse gastric lipomatosis is rarer, and pyloric involvement may cause bleeding or intermittent gastric outlet obstruction. We report a pylorus-spanning lipomatosis treated definitively by laparoscopic distal gastrectomy.

**Presentation of case:**

A 75-year-old man presented with melaena and post-prandial epigastric discomfort. Endoscopy revealed a broad-based submucosal mass at the pyloric canal intermittently prolapsing into the duodenal bulb with a small healing ulcer. computed tomography (CT) showed an 8.1 × 2.7 × 3.8 cm multiseptated fat-density mass traversing the pylorus with luminal narrowing. Positron emission tomography-CT demonstrated no abnormal hypermetabolism. Because of recurrent symptoms and trans-pyloric extension, laparoscopic distal gastrectomy with Billroth II reconstruction was performed. Histopathology confirmed diffuse lipomatosis of mature adipocytes without atypia. Recovery was uneventful; 1-month and 2-year imaging showed durable patency without recurrence.

**Clinical discussion:**

Gastric lipomatosis is rare. Treatment options include endoscopic submucosal dissection and laparoscopic–endoscopic cooperative approaches such as laparoscopic–endoscopic cooperative surgery, non-exposure endoscopic–laparoscopic cooperative surgery (NEWS), and CLEAN-NET (combination of laparoscopic and endoscopic approaches to neoplasia with non-exposure technique). In pylorus-spanning diffuse lipomatosis, however, distal gastrectomy remains among the most reliable strategies for definitive symptom control.

**Conclusion:**

In symptomatic, pylorus-spanning gastric lipomatosis, laparoscopic distal gastrectomy offered dependable, durable relief of obstruction.

## Introduction

Gastric lipomas constitute a small proportion of benign gastric neoplasms; most are incidental, but larger lesions or those in the distal stomach and pyloric region can ulcerate, bleed, or precipitate intermittent gastric outlet obstruction^[1–3]^. On computed tomography (CT), homogeneous fat attenuation with delicate internal septations strongly favors a lipomatous lesion^[2,4]^. Diffuse gastric lipomatosis – multifocal or infiltrative adipocytic proliferation within the gastric wall – is exceptionally uncommon and may span the antral–pyloric region, complicating endoluminal management^[5,6]^. This case report has been reported in line with the SCARE checklist^[7]^.

**Highlights**

- Pylorus‑spanning gastric lipomatosis is rare.
- Diagnosis is feasible with endoscopy and computed tomography.
- Patients may present with bleeding or gastric outlet obstruction.
- Endoscopic resection suits focal lesions in selected settings.
- Diffuse trans‑pyloric disease favors distal gastrectomy for durability.

## Case presentation

### Patient information

A 75-year-old man experienced 3 days of melena and several weeks of early satiety and post-prandial epigastric heaviness. He first attended an emergency department, where hemodynamic parameters were stable and proton-pump inhibitor therapy was initiated. Persistent symptoms led to review in an internal medicine clinic and referral to gastroenterology. After endoscopic and radiologic evaluation demonstrated a pylorus-spanning lesion associated with intermittent outlet compromise, he was referred to general surgery for operative management. On surgical assessment, there was mild epigastric tenderness without peritoneal signs or weight loss. Medical and family histories were non-contributory; there were no known drug allergies.

### Diagnostic assessment

**Figure 1.**
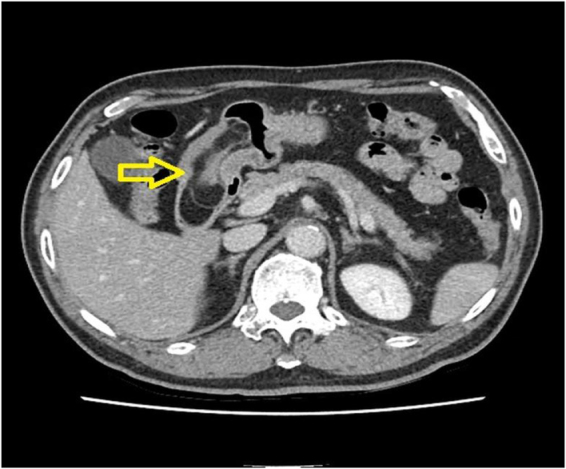
Pre-operative CT, multiseptated fat-density mass (8.1 × 2.7 × 3.8 cm) centered on the distal antrum/pyloric channel, traversing into the duodenal bulb with segmental luminal narrowing.

**Figure 2.**
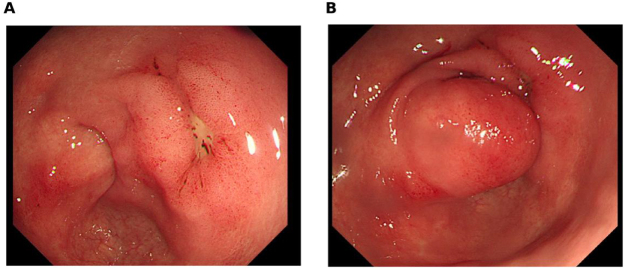
Pre-operative endoscopy. (A) Broad-based submucosal mass at the pyloric canal (pyloric ring view). (B) Transient prolapse of the lesion into the duodenal bulb with retraction.

Contrast-enhanced CT demonstrated an 8.1 × 2.7 × 3.8 cm multiseptated fat-density mass centered on the distal antrum/pyloric channel, extending into the duodenal bulb with segmental luminal narrowing (Fig. 1). Esophagogastroduodenoscopy showed a smooth, broad-based submucosal tumor at the pyloric canal that intermittently prolapsed into the duodenal bulb; a 0.7-cm healing ulcer was present on the opposing wall (Fig. 2). Positron emission tomography–computed tomography (PET-CT) revealed no abnormal hypermetabolic uptake.

### Differential diagnosis

The differential diagnosis included gastric lipoma/lipomatosis; well-differentiated liposarcoma (considered less likely given uniform fat attenuation, absence of nodular soft-tissue components, and PET-CT negativity, although histology is definitive); and other subepithelial lesions such as gastrointestinal stromal tumor with fatty degeneration, ectopic pancreas, or inflammatory fibroid polyp, which were less consistent with the imaging phenotype and antral–pyloric location^[2–4]^.

### Therapeutic intervention

Given recurrent symptoms, trans-pyloric extent, and a broad sessile base precluding safe endoscopic enucleation, the patient underwent laparoscopic distal gastrectomy. Intraoperatively, the duodenal bulb was short and edematous with limited mobility after resection of the pylorus-spanning lesion. To avoid a high-tension gastroduodenostomy with a small-caliber duodenal stump, an antecolic Billroth-II gastrojejunostomy was performed to ensure a tension-free, adequately wide anastomosis. Estimated blood loss was minimal; no drains were placed.

### Pathological results

**Figure 3.**
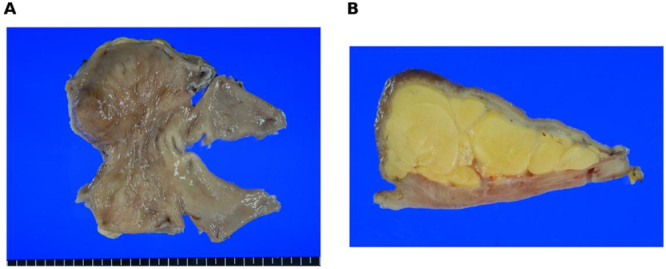
Pathological staining for gastric lipomatosis (A) submucosal expansion by lobulated mature adipose tissue (H&E staining, 40×), (B) uniform mature adipocytes without cytologic atypia or lipoblasts; no necrosis or mitoses (H&E staining, 200×).

**Figure 4.**
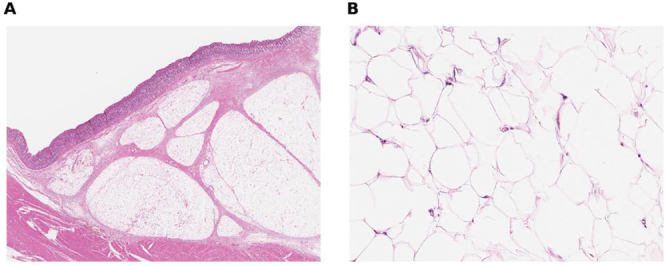
Postoperative CT. (A) One month after – restored luminal patency without residual fat-density lesion. (B) Two years after – no radiologic recurrence 2 years after distal gastrectomy.

Grossly, the distal antrum/pyloric channel showed a broad-based, yellow lobulated lesion; on the cut surface, lobulated adipose tissue with thin septa was evident (Fig. 3). Histology revealed mature adipocytes in lobules separated by delicate fibrous septa without atypia, lipoblasts, necrosis, or increased mitotic activity (Fig. 4). Resection margins were free of lesion, consistent with diffuse gastric lipomatosis rather than well-differentiated liposarcoma.

### Outcome and follow-up

**Figure 5.**
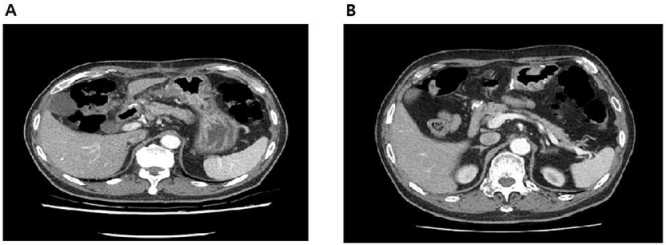
Gross pathology (A) resected distal antrum/pyloric channel showing broad-based bulging lesion with mucosal ulcer bed, (B) cut surface with yellow lobulated adipose tissue and thin fibrous septa without nodular soft-tissue component.

The postoperative course was uncomplicated with early diet advancement. One-month CT confirmed restored luminal patency; at 2 years, there was no radiologic recurrence and postoperative complications (Fig. 5).

## Discussion

Gastric lipomas are rare, and diffuse gastric lipomatosis is rarer still. Symptomatic presentations most commonly involve bleeding or episodic gastric outlet obstruction, particularly when lesions occupy the antral–pyloric region and intermittently prolapse into the duodenal bulb^[1–3]^. Diagnostic confidence is high when CT demonstrates homogeneous fat attenuation (approximately −80 to −120 Hounsfield units) with thin septa and without soft-tissue nodularity; routine mucosal biopsies are often non-diagnostic because pathology is submucosal^[2,4]^. Endoscopy establishes location and assesses mucosal integrity; when obtained, endoscopic ultrasonography typically depicts a hyperechoic, submucosal lesion. PET-CT is usually non-avid in benign lipomatous lesions and can help exclude liposarcoma, although histopathology remains definitive where doubt persists^[2–4]^. Management should be anatomy-based. Observation is reasonable for small, asymptomatic lesions. For symptomatic disease or complications (ulceration, bleeding, and obstruction), intervention is warranted. Endoscopic resection – most commonly endoscopic submucosal dissection – achieves en-bloc removal with excellent outcomes in selected focal submucosal lipomas located away from the pylorus and with an adequate submucosal cushion^[8–10]^. In contrast, diffuse lipomatosis or trans-pyloric involvement reduces the likelihood of complete endoluminal clearance and increases the risks of luminal deformity or restenosis after debulking, favoring surgical resection^[2,3,6]^. Laparoscopic–endoscopic cooperative surgery integrates endoscopic mucosal marking or resection with laparoscopic full-thickness control; non-exposure endoscopic–laparoscopic cooperative surgery (NEWS) and combination of laparoscopic and endoscopic approaches to neoplasia with non-exposure technique (CLEAN-NET) were developed to minimize peritoneal contamination^[11–13]^. These approaches are well described for submucosal gastric tumors – particularly gastrointestinal stromal tumors – but experience in diffuse lipomatosis is limited. Where the lesion spans the pylorus or is infiltrative, distal gastrectomy remains the most dependable approach for anatomical correction and durable symptom resolution^[2,3,6]^. Given the lesion’s trans-pyloric extension, broad sessile base, and recurrent obstructive symptoms, distal gastrectomy was indicated. Although Billroth I is preferred when a low-tension gastroduodenostomy is anatomically feasible, intraoperative findings of a shortened, relatively stiff duodenal bulb made a tension-free gastroduodenostomy uncertain and at risk for late stenosis. We therefore performed an antecolic Billroth II gastrojejunostomy to secure a wide, tension-free outflow tract; the postoperative course was uneventful, and CT at 1 month and 2 years confirmed durable radiologic remission.

## Conclusion

Pylorus-spanning gastric lipomatosis is an uncommon but important cause of intermittent gastric outlet obstruction. When anatomy predicts incomplete endoscopic clearance – particularly with trans-pyloric, diffuse disease – laparoscopic distal gastrectomy offers reliable, durable resolution of symptoms.

## Data Availability

The data that support the findings of this study are available from the corresponding author upon reasonable request.
